# Common Gamma Chain Cytokines Promote Rapid *In Vitro* Expansion of Allo-Specific Human CD8^+^ Suppressor T Cells

**DOI:** 10.1371/journal.pone.0028948

**Published:** 2011-12-14

**Authors:** Yuming Yu, Jennifer R. Zitzner, Josetta Houlihan, Nancy Herrera, Luting Xu, Joshua Miller, James M. Mathew, Anat R. Tambur, Xunrong Luo

**Affiliations:** 1 Comprehensive Transplant Center, Northwestern University Feinberg School of Medicine, Chicago, Illinois, United States of America; 2 Department of Organ Transplantation, Nanfang Hospital, Southern Medical University, Guangzhou, Guangdong, China; 3 Department of Medicine, Northwestern University Feinberg School of Medicine, Chicago, Illinois, United States of America; University Medical Center Freiburg, Germany

## Abstract

Human CD8^+^ regulatory T cells, particularly the CD8^+^CD28^−^ T suppressor cells, have emerged as an important modulator of alloimmunity. Understanding the conditions under which these cells are induced and/or expanded would greatly facilitate their application in future clinical trials. In the current study, we develop a novel strategy that combines common gamma chain (γc) cytokines IL-2, IL-7 and IL-15 and donor antigen presenting cells (APCs) to stimulate full HLA-mismatched allogeneic human CD8^+^ T cells which results in significant expansions of donor-specific CD8^+^CD28^−^ T suppressor cells *in vitro*. The expanded CD8^+^CD28^−^ T cells exhibit increased expressions of CTLA-4, FoxP3, and CD25, while down-regulate expressions of CD56, CD57, CD127, and perforin. Furthermore, these cells suppress proliferation of CD4^+^ T cells in a contact-dependent and cytokine-independent manner. Interestingly, the specificity of suppression is restricted by the donor HLA class I antigens but promiscuous to HLA class II antigens, providing a potential mechanism for linked suppression. Taken together, our results demonstrate a novel role for common γc cytokines in combination with donor APCs in the expansion of donor-specific CD8^+^CD28^−^ T suppressor cells, and represent a robust strategy for *in vitro* generation of such cells for adoptive cellular immunotherapy in transplantation.

## Introduction

Allogeneic organ transplantation has emerged as the current best therapeutic option for selected patients with end stage organ failure. Tolerance induction is highly desirable in this population, because long-term immunosuppressive therapy is associated with significant risks for opportunistic infections, degenerative and metabolic diseases, as well as malignancies. Regulatory T cell (Treg) based therapy has emerged as a promising means for immunomodulation to achieve transplant tolerance. Great interest has been focused on CD4^+^ Tregs [1], and cell therapies using adoptive transfer of *in vitro* generated CD4^+^ Treg cells have demonstrated promising efficacy for immunomodulation in animal models of allogeneic transplantation as well as in clinical trials of human allogeneic bone marrow transplantation for the control of graft versus host disease [2], [3], [4]. However, evidence also suggests that CD8^+^ Tregs may play an important regulatory role in transplant tolerance [5], [6], [7], [8], in addition to possible immunomodulatory roles in autoimmune disorders [9], [10], cancers [11] and aging [12]. Several subsets of CD8^+^ Tregs have been observed. Natural CD8^+^ Tregs have been reported to be CD8^+^CD25^+^, CD8^+^CD122^+^, or CD8^+^CXCR3^+^ in different systems [13], [14], [15]. Induced CD8^+^ Tregs have also been reported to bear various phenotypic characteristics, such as CD28^−^, CD56^+^, CD57^+^, CTLA4^+^, CD103^+^, CD25^+^Foxp3^+^ or LAG3^+^CCL4^+^ [5], [16], [17], [18], [19]. These natural or induced CD8^+^ Tregs exert their suppressive function by cell-cell contact [5] or by producing soluble cytokines [15], [17], and in some circumstances by inducing tolerogenicity in antigen presenting cells (APCs) [20].

Among the various CD8^+^ Tregs, CD8^+^CD28^−^ T suppressor cells have emerged as an important modulator of alloimmunity. Human *allo-specific* CD8^+^CD28^−^ cells have been shown to emerge after repeated stimulation with allogeneic APCs with supplemental recombinant human IL-2 *in vitro* [5]. These cells have subsequently been termed “Ts cells”. Similarly, *autoantigen-specific* CD8^+^ Tregs including CD8^+^CD28^−^ cells have been reported to be induced *in vitro* via stimulation of peripheral T cells obtained from patients with systemic lupus erythematosus (SLE) in the presence of a combination of common gamma chain (γc) cytokines IL-2, IL-7 and IL-15 [21]. These conditions under which CD8^+^CD28^−^ cells are induced are distinct from those in which CD4^+^ Tregs are induced, in that the latter frequently require the presence of tolerance promoting agents such as TGF-β, IL-10, or rapamycin in the culture [22], [23], [24], [25]. While the mechanisms by which CD8^+^CD28^−^ cells are induced under such apparently non-tolerance promoting conditions remain unclear, the induced CD8^+^CD28^−^ cells nevertheless exhibit potent antigen-specific suppressive capacities [5], [21]. The γc cytokines have also been reported to induce stable loss of CD28 expression in actively dividing CD8^+^CD28^+^ T cells [26], [27], thereby promoting the generation of a preponderance of CD8^+^CD28^−^ T cells. We therefore questioned if γc cytokines in combination with donor APCs may be used to expand *donor-specific* CD8^+^CD28^−^ T suppressor cells in large numbers *in vitro*, and if so, what the mechanisms of donor-specific suppression are.

In this report, we utilized APCs and T cells variably matched for human leukocyte antigen (HLA) class I or class II to study the ability and mechanisms of suppression of the CD8^+^CD28^−^ T cells generated *in vitro* by this novel approach of combining allogeneic APCs plus γc cytokines. Our findings indicate that large numbers of CD8^+^CD28^−^ T cells with potent donor-specific suppressive capacity can be effectively generated *in vitro* using this approach. Furthermore, their suppressive capacity is contact-dependent and is restricted only by the donor HLA class I antigens but promiscuous to HLA class II antigens. To our knowledge, this is the first report demonstrating a role of combination γc cytokines in the *in vitro* generation of large numbers of human donor-specific CD8^+^CD28^−^ suppressor cells. Findings from this study may have important implications for designing future Treg based therapies for prevention of rejection in human organ or tissue transplantation.

## Results

### Donor APCs plus γc cytokines induce rapid expansion of CD8^+^CD28^−^ T cells in culture

**Figure 1:**
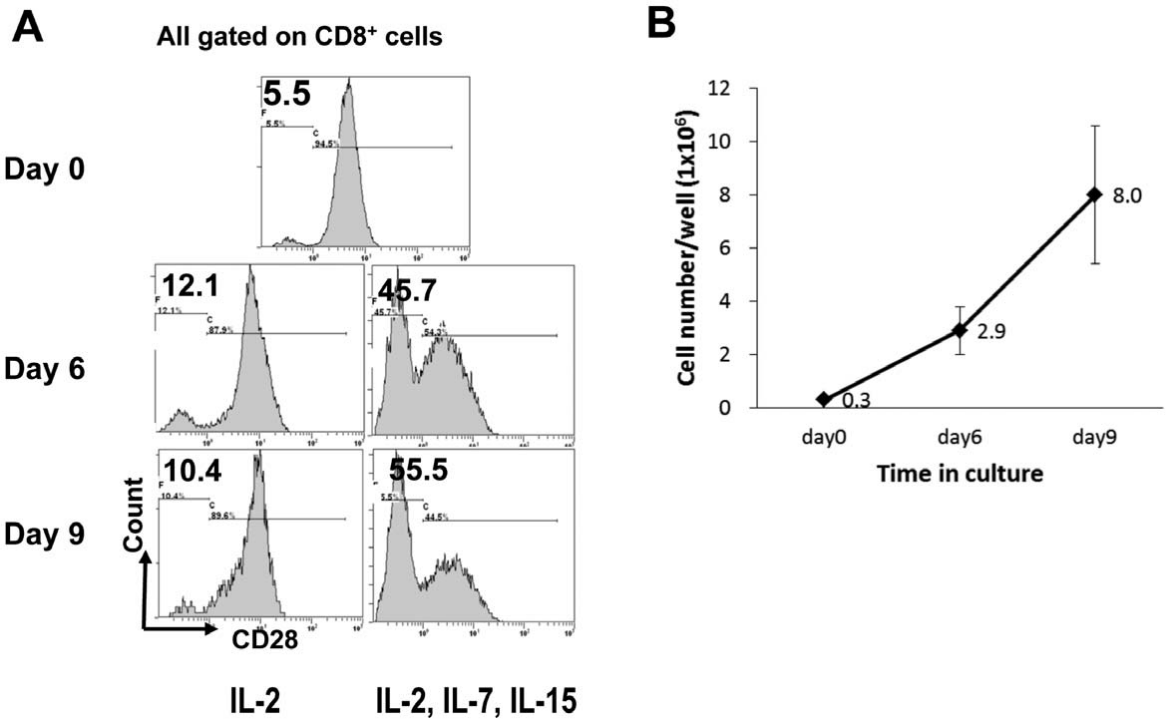
Donor APCs plus γc cytokines induce rapid expansion of CD8^+^CD28^−^ T cells in culture. Freshly purified CD8^+^ T cells (2×10^6^ per well) from healthy volunteers were stimulated by HLA-A, B and DR mismatched allogeneic APCs (1×10^6^ per well) for 9 days in 24-well plates supplemented with IL-2 alone or a combination of IL-2, IL-7 and IL-15. *A*, Expression of CD28 on CD8^+^ cells over time. Histograms were gated on the CD8^+^ population. *B*, Expansion of cell numbers of the CD8^+^CD28^−^ population over time by donor APC plus γc cytokines stimulation. The numbers of the CD8^+^CD28^−^ cells per well of 24-well culture plates were calculated for day 0, 6 and 9. Data shown in *A* are representative of three independent experiments. Data shown in *B* is the average of three independent experiments.

The CD28^−^ cell population accounts for a small fraction of freshly isolated human CD8^+^ T cells from PBMCs (5.5%, Figure 1A, top panel, Day 0). Fresh CD8^+^ T cells were stimulated with HLA-A, -B and -DR complete-mismatched APCs in the presence of IL-2 alone or a combination of γc cytokines IL-2, IL-7 and IL-15 as described in *Materials and Methods* [21]. As shown in Figure 1A bottom panels (Day 6, Day 9), culture in the presence of IL-2 alone allowed only minimal increase of the CD28^−^ population over time. In contrast, culture in the presence of a combination of γc cytokines IL-2, IL-7 and IL-15 significantly increased this population from 5.5% of all CD8^+^ cells on day 0 to 45.7% (51.5+/−8.5%) on day 6, and 55.5% (58.5+/−7.2%) on day 9 (Figure 1*A*), likely representing down-regulation of cell surface CD28 during the *in vitro* cell activation as previously described [26], [27]. The total number of CD8^+^CD28^−^ recovered at the end of the 9 day culture increased by ∼26.7+/−7.5 fold (Figure 1*B*). The most dramatic increase of this population was observed when IL-15 was added to the culture, with moderate effect observed with IL-7 on the expansion of this population (data not shown).

### *In vitro* expanded CD8^+^CD28^−^ T cells suppress CD4^+^ T cells proliferation in a donor-specific manner

**Figure 2:**
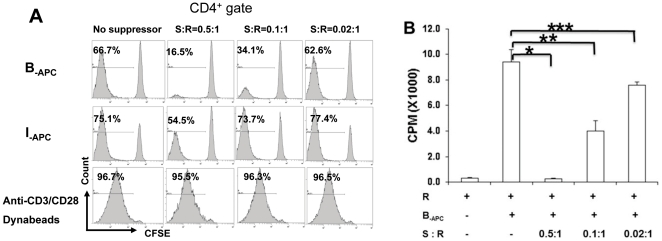
*In vitro* expanded CD8^+^CD28^−^ T cells suppress CD4^+^ T cells proliferation in a donor-specific manner. CD8^+^ T cells from donor A were stimulated with APCs from donor B plus γc cytokines for 9 days, followed by enrichment of the CD8^+^CD28^−^ population as described in *Materials and Methods*. 5×10^4^ CFSE labeled purified responder CD4^+^ T cells (R) from donor A were stimulated with 5×10^4^ APCs from donor B (B-_APC_) or from an indifferent donor (I-_APC_, HLA-A, B and DR fully mismatched with donor B), or 1×10^4^ anti-CD3/CD28 coated Dynabeads in triplicates in 96-well plates. The *in vitro* expanded CD8^+^CD28^−^ T cells were added as putative suppressors (S) at S∶R ratios of 0.5∶1, 0.1∶1 and 0.02∶1 (with the cell number of “R” kept constant). Proliferation of the CD4^+^ cells was measured by CFSE dilution (*A*) or by ^3^H thymidine uptake (*B*). Data shown are representative of three independent experiments. * *P* = 0.002; ** *P* = 0.009; *** *P* = 0.008.

CD8^+^CD28^−^ T cells were generated from individual A by culturing with allogeneic APCs from individual B (designated as B-_APC_) in the presence of the γc cytokine combination as described above. After 9 days of culture, the resulting CD8^+^CD28^−^ T cells were isolated (purity>95%) and used as suppressors (S) to test for their suppressive capacity in mixed lymphocyte reactions (MLRs) using CD4^+^ T cells from individual A (designated as A-T4) as responders (R). Suppressor to responder ratios (S∶R) of 0.5∶1, 0.1∶1 and 0.02∶1 were tested (with the cell number of “R” kept constant). As shown in Figure 2*A*, when APCs from the original priming stimulator (B-_APC_) were used as stimulators of the MLRs, proliferation of A-T4 (measured by CFSE dilution) was markedly suppressed in a dose-dependent manner by the *in vitro* expanded CD8^+^CD28^−^ T cells. Suppression was also confirmed by using ^3^H-thymidine incorporation as a read-out (Figure 2*B*). In contrast, when APCs from an HLA-A, -B and -DR mismatched indifferent stimulator (I-_APC_) were used as stimulators of the MLRs, CD8^+^CD28^−^ cells exhibited only marginal suppression at the highest S∶R ratio tested (0.5∶1, shown in Fig. 2*A*, middle panels). No suppression could be demonstrated when anti-CD3/CD28 coated Dynabeads were used for stimulating the CD4^+^ T cells (Figure 2*A*, bottom panels). Taken together, our results indicate that the CD8^+^CD28^−^ T cell generated from donor APCs plus γc cytokine-driven *in vitro* expansion suppress CD4^+^ T cells proliferation *only* in a donor-specific manner.

### Suppression by the *in vitro* expanded CD8^+^CD28^−^ T cells is restricted by donor HLA class I antigens but promiscuous to HLA class II antigens

**Figure 3:**
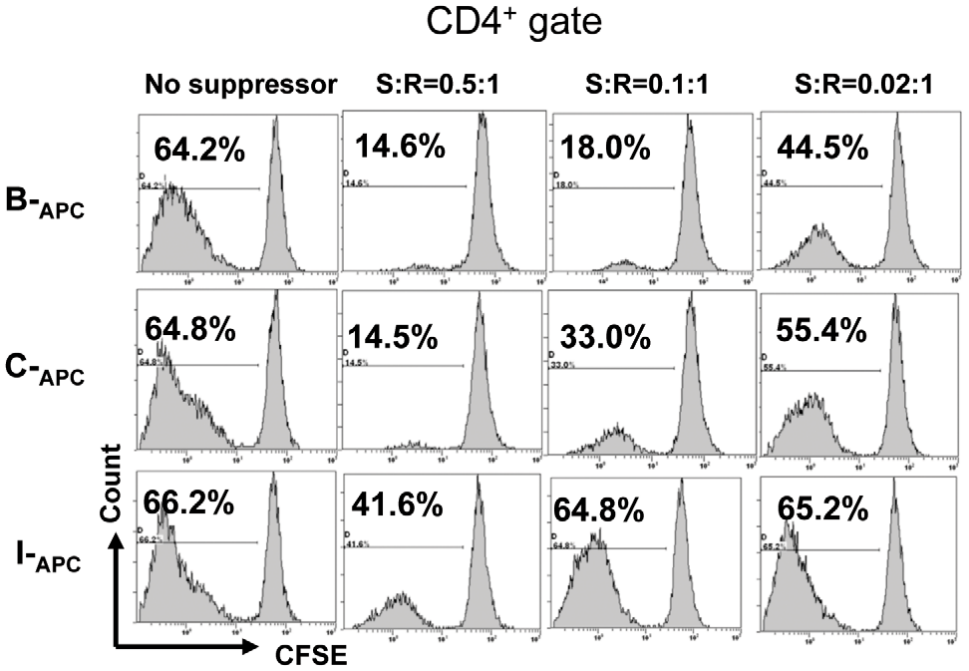
Suppression by the *in vitro* expanded CD8^+^CD28^−^ T cells is restricted by donor HLA class I antigens. Suppression assays were set up as described in Figure 2, with the exception that the stimulator APCs for CD4^+^ proliferation were from either the priming donor (B-_APC_), an indifferent donor (I-_APC_, HLA-A, B and DR fully mismatched with donor B), or a partially matched donor (C-_APC_, who shared identical HLA class I, but not class II antigens, with donor B). Proliferation of the CD4^+^ cells was measured by CFSE dilution. Data shown are representative of three independent experiments using three pairs of donors B, C and I. Table 1 provides one example of the HLA class I and class II antigen typing of individuals B, C, and I used for one of three such experiments.

To determine whether the donor specificity observed in the above suppression of proliferation was restricted via recognition of HLA class I antigens only, we examined the ability of the CD8^+^CD28^−^ T cells generated from culturing with B-_APC_ to suppress proliferation stimulated by APCs from a third-party donor (C-_APC_) who shared identical HLA class I but not class II antigens with individual B. The ability of suppressing C-_APC_ driven proliferation was compared with that of suppressing I-_APC_ driven proliferation (I-_APC_ were from an indifferent individual who was mismatched with individual A for *both* HLA class I and class II antigens). Table 1 provides one example of the HLA class I and class II antigen typing of individuals A, B, C, and I used for one of three such experiments. As shown representatively in Figure 3, proliferation of A-T4 was profoundly suppressed when B-_APC_ was used as the stimulator as expected. Interestingly, a significant suppression was also observed, albeit at a slightly reduced level, when C-_APC_ was used as the stimulator. In contrast, suppression was almost completely lost when I-_APC_ was used as the stimulator. These data support the hypothesis that suppression by the *in vitro* expanded CD8^+^CD28^−^ T cells is mediated via specific interactions between the T cell receptor (TCR) on the CD8^+^ cells and donor HLA class I molecules on the APCs, and such interactions can lead to suppression of CD4^+^ cells with TCRs specific for HLA class II molecules co-expressed on the same interacting APCs (the C-_APC_ in this case) but different from those expressed on the original priming APCs (the B-_APC_ in this case). This finding corroborates with the previous observation that CD8 suppressor cells are capable of exerting linked suppression via interactions with APCs [28].

**Table 1:** Representative HLA typing of donors used for experiments demonstrating HLA class I antigen specificity shown in Figure 3.

Cell type	HLA-A		HLA-B		HLA-DR	
**A** **(CD8^+^CD28^−^ cells or A-T4**	11	-	18	35	1	7
**B-_APC_**	1	2	8	44	4	15(2)
**C-_APC_**	1	2	8	44	3	12(5)
**I-_APC_**	24	26	7	61	9	10

The HLA typing of donors for CD8^+^ T cells for generation of CD8^+^CD28^−^ suppressor cells, the priming B-_APC_ cells, the partially matched C-_APC_ cells (which shared identical HLA-A, -B, but not HLA-DR, with B-_APC_), or the fully mismatched I-_APC_ cells (HLA-A, B and DR fully mismatched with B-_APC_) are shown. Two additional sets of donors A, B, C, and I were used for repeating this experiment for a total of three independent times.

### Suppression by the *in vitro* expanded CD8^+^CD28^−^ T cells is contact dependent but IFN-γ or TGF-β independent

**Figure 4:**
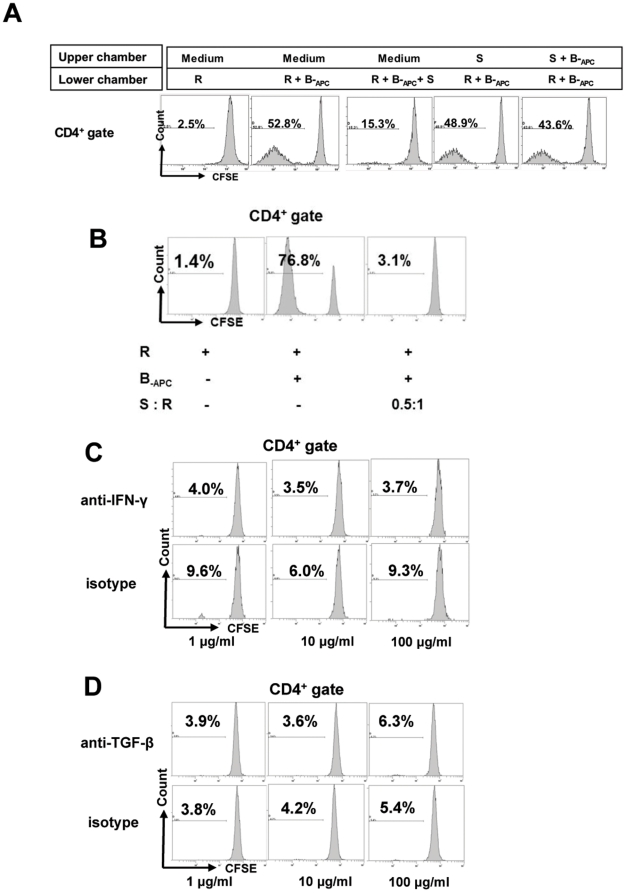
Suppression by the *in vitro* expanded CD8^+^CD28^−^ T cells is contact dependent but IFN-γ or TGF-β independent. Suppression assays were set up as described in Figure 2 at an S∶R ratio of 0.5∶1. Proliferation of the CD4^+^ cells was measured by CFSE dilution. *A*, Transwell assays: the lower chambers of 24-well transwell plates were plated with CFSE-labeled responder cells (3×10^5^ of R) and stimulator cells (3×10^5^ of B-_APC_), and the *in vitro* expanded CD8^+^CD28^−^ T cells were added either in the lower chamber to allow cell-cell contact or in the upper chamber to prevent cell-cell contact. Data shown are representative of three independent experiments. *B*, Control suppression without any blocking antibodies. *C*, Suppression in the presence of anti-human IFN-γ antibody or its isotype control antibody at concentrations of 1 µg/ml, 10 µg/ml and 100 µg/ml. *D*, Suppression in the presence of anti-human TGF-β antibody or its isotype control antibody at concentrations of 1 µg/ml, 10 µg/ml and 100 µg/ml. Data shown for *B* and *C* are representative of three independent experiments. Abbreviations used for *A*–*D*: “R”: CD4^+^ responder cells from donor A; “B-_APC_”: APC stimulators from donor B; “S”: the *in vitro* expanded CD8^+^CD28^−^ T cells.

The role of cell-cell contact in suppression was examined using transwell assays as shown in Figure 4*A*. The lower chamber was plated with CFSE-labeled responder cells (R) and stimulator cells (B-_APC_), and the *in vitro* expanded CD8^+^CD28^−^ T cells (S) were added either in the lower chamber to allow cell-cell contact or in the upper chamber to prevent cell-cell contact. As shown in Figure 4*A*, suppression of proliferation by CD8^+^CD28^−^ T cells was abolished when these cells were plated in the upper chamber of the transwells, indicating that suppression by these cells requires cell-cell contact between the CD8^+^CD28^−^ T cells, stimulator APCs and/or the responder CD4^+^ T cells. This finding is consistent with the characteristic donor HLA class I restriction of the suppressive activity of the *in vitro* expanded CD8^+^CD28^−^ T cells observed in Figure 3.

Several reports have implicated TGF-β [13], [29], [30] and IFN-γ [8], [31], [32] in the mechanisms of regulation by CD8^+^ suppressor cells. To test for a potential role of TGF-β or IFN-γ in the suppressive capacity of the *in vitro* expanded CD8^+^CD28^−^ T cells in our system, anti-human IFN-γ or anti-human TGF-β antibody was added to the suppression assays described above (baseline suppression without the blocking antibodies is shown in Figure 4*B*). As shown in Figure 4*C* and Figure 4*D*, neither anti-IFN-γ nor anti-TGF-β antibodies at increasing concentrations affected suppression by CD8^+^CD28^−^ T cells.

### *In vitro* expanded CD8^+^CD28^−^ T cells do not exhibit cytotoxicity

**Figure 5:**
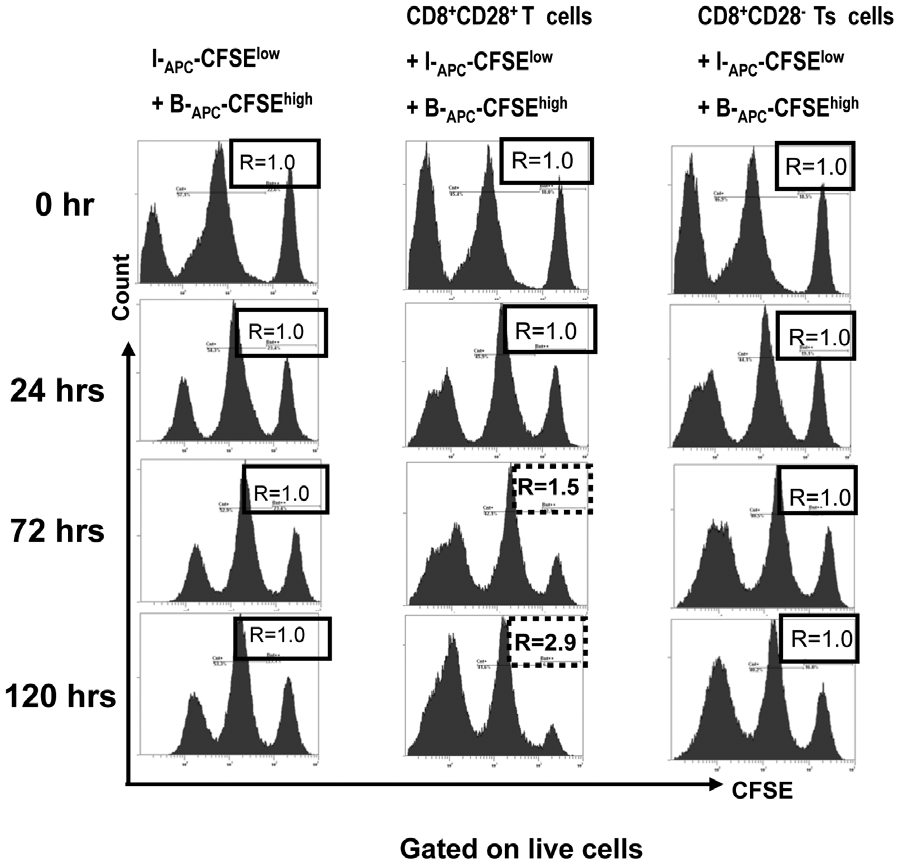
Cytotoxicity does not contribute to the suppression of proliferation by the *in vitro* expanded CD8^+^CD28^−^ T cells. A CFSE based cytotoxicity assay was set up as follows: target cells were the original priming APCs (for generating the CD8^+^CD28^−^ T cells) labeled with a high concentration (2.0 µM) of CFSE (B-_APC_-CFSE^high^), or APCs from an HLA-A, -B, and -DR mismatched indifferent individual labeled with a low concentration (0.2 µM) of CFSE (I-_APC_-CFSE^low^). A 1∶1 mixture of I-_APC_-CFSE^low^ and B-_APC_-CFSE^high^ cells were plated either by themselves (left panels), or with the same number of control (CD8^+^CD28^+^, middle panels) or putative (CD8^+^CD28^−^, right panels) effector cells in 96 U-bottom plates in triplicates. At 24, 72, and 120 hr of culture, cells were collected and analyzed by FACS to enumerate CFSE^low^ and CFSE^high^ cells. The ratio of cell numbers of I-_APC_-CFSE^low^ over B-_APC_-CFSE^high^ was calculated as “R” for all time points. An increase in the value of R indicates specific killing of B-_APC_-CFSE^high^ cells by the effector cells, whereas a stable value of R at 1.0 indicates no specific killing of B-_APC_-CFSE^high^ cells by the effector cells. Data shown are representative of two independent experiments.

To test whether cytotoxicity towards the stimulating APCs contributes to the suppression of donor-specific proliferation observed above, we assessed the cytotoxicity of CD8^+^CD28^−^ T cells using a CFSE-based assay [33]. We designed this assay to specifically determine the ability of the CD8^+^CD28^−^ T cells to lyse allogeneic donor APCs used as stimulators in the above MLRs. In this assay, “effector cells” were the CD8^+^CD28^−^ T cells generated by the donor APC plus γc cytokine-driven *in vitro* cultures as above. The CD8^+^CD28^+^ T cells generated by the same cultures were used as control effector cells because of their known cytotoxicity [5], [34], [35]. Target cells were either the original priming APCs which were labeled with a high concentration of CFSE (B-_APC_-CFSE^high^), or control APCs from an HLA-A, -B, and -DR mismatched indifferent individual which were labeled with a low concentration of CFSE (I-_APC_-CFSE^low^). A 1∶1 mixture of I-_APC_-CFSE^low^ and B-_APC_-CFSE^high^ cells were plated either by themselves or with the same number of the putative (CD8^+^CD28^−^) or control (CD8^+^CD28^+^) effector cells. At 24, 72, and 120 hr of culture, cells were collected and analyzed by FACS to enumerate CFSE^low^ and CFSE^high^ cells. The ratio of cell numbers of I-_APC_-CFSE^low^ over B-_APC_-CFSE^high^ (shown as “R” in Figure 5) over time provided a measure of specific cytolytic effect of the putative effector cells on these two target populations. As shown in Figure 5 left panels, minimal spontaneous lysis was observed as the ratio R remained stable over time in the absence of any effector cells. When the CD8^+^CD28^+^ T cells were added as effector cells (Figure 5, middle panels), R increased to 1.5 at 72 hrs and 2.9 at 120 hrs, demonstrating that B-_APC_-CFSE^high^ cells were specifically killed by the CD8^+^CD28^+^ T cells. In contrast, when the CD8^+^CD28^−^ T cells were added as effector cells, R remained unchanged over time, indicating that the CD8^+^CD28^−^ T cells exhibit no specific cytolysis towards the B-_APC_ cells (Figure 5, right panels).

### Phenotypic characteristics of the *in vitro* expanded CD8^+^CD28^−^ T cells

**Figure 6:**
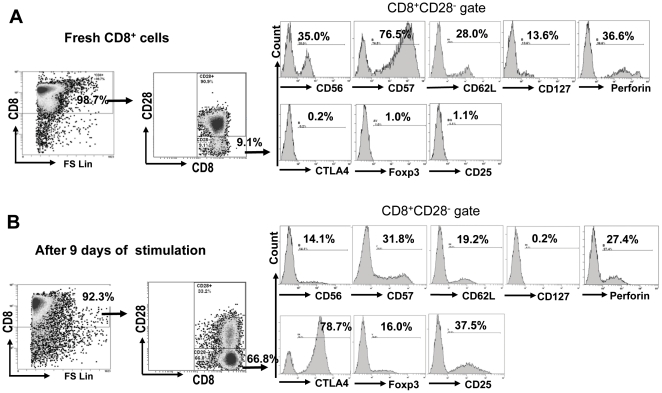
Phenotypic characteristics of the *in vitro* expanded CD8^+^CD28^−^ T cells. CD8^+^ T cells freshly isolated from healthy volunteers (*A*) or after *in vitro* stimulation with donor APCs plus γc cytokines for 9 days (*B*) were analyzed for cell surface or intracellular markers by multichromatic flow cytometry. Date shown are representative of three independent experiments.

Phenotypic characteristics of CD8^+^CD28^−^ T cells were examined before and after donor APCs plus γc cytokine-driven *in vitro* expansion to determine if reported markers of CD8 Tregs such as CTLA-4, FoxP3, CD25, CD56 and CD57 [5], [16], [17], [18], [19] were also expressed by the CD8^+^CD28^−^ T cells generated by our protocol. As shown in Figure 6*A*, among freshly isolated CD8^+^ T cells prior to *in vitro* stimulation, CD8^+^CD28^−^ T cells comprised a small fraction. At baseline, they expressed CD56 (35.0%), CD57 (76.5%), CD62L (28.0%), CD127 (13.6%), and perforin (36.6%), but exhibited minimal expressions of CTLA-4, FoxP3, CD25 (Figure 6*A*). Following donor APCs plus γc cytokine-driven expansion, CD28^−^ cells became the predominant population among CD8^+^ cells, and they significantly up-regulated expressions of CTLA-4 (78.7%), FoxP3 (16.0%), and CD25 (37.5%) compared with basline, while down-regulated expressions of CD56, CD57, CD62L, CD127, and perforin (Figure 6*B*). While the exact percentages of these markers varied between the CD8^+^CD28^−^ cells from different individuals (data not shown), the trend of up- or down-regulations before and after stimulation in cultures remained the same (1 of 3 representative experiments is shown in Figure 6).

## Discussion

In this study, we reported the novel finding that large numbers of donor-specific human CD8^+^CD28^−^ T suppressor cells can be generated *in vitro* using a combination of donor APCs and γc cytokines stimulated proliferation. Common γc cytokines IL-2, IL-7 and IL-15 play critical roles in T cell homeostasis [36]. Recent reports have demonstrated that IL-15 can promote the generation and proliferation of CD8^+^ memory T cells when stimulated by anti-CD3 and anti-CD28 antibodies [12], [26]. In contrast, a combination of γc cytokines including IL-2, IL-7 and IL-15 has been used to expand CD8^+^Foxp3^+^ Treg *in vitro* from autoimmune patients with SLE who have undergone autologous bone marrow transplant [21]. Human allo-specific CD8^+^CD28^−^ suppressor T cells have been reported to be generated by two rounds of stimulation by allogeneic APCs in which IL-2 was the only cytokine added during the last four days of cultures [20]. These cells have been termed as Ts cells. Here, we demonstrated that the combination of IL-2, IL-7 and IL-15, compared with IL-2 alone, was superior in inducing significant expansions of allo-specific CD8^+^CD28^−^ cells in cultures activated by donor APC stimulation (Figure 1*A, 1B*), and that the expanded CD8^+^CD28^−^ cells were highly functionally suppressive. The role of each individual cytokine in the expansion versus inducing the “suppressive” characteristics of this cell population remains to be further elucidated.

The phenotypic and functional characteristics of the CD8^+^CD28^−^ T cells generated in our protocol with a combination of γc cytokines closely resemble those of previously described Ts cells [19], [20]. They both up-regulate CTLA-4, FoxP3 and CD25, and have variable degrees of down-regulation of CD62L during expansion; possess minimal cytotoxicity; and exhibit suppressive capacity in a contact dependent fashion. We further demonstrated that our *in vitro* expanded CD8^+^CD28^−^ T cells exert linked suppression of CD4 T cells of a different HLA class II specificity as long as the HLA class II molecules are co-expressed with the priming HLA class I molecules on the same APCs. This has also been previously indirectly demonstrated as a characteristic of Ts cells [5]. The ability to generate large numbers of functionally suppressive donor-specific CD8^+^CD28^−^ T cells that closely mimic Ts cells with this novel approach of using common γc cytokines thus has significant potential for facilitating future clinical use of Treg cell-based immunotherapy for tolerance induction in transplantation.

Several mechanisms of suppression by CD8 suppressor cells have been described. These include direct cytotoxicity [37], secretion of inhibitory cytokines [32], [38], down-regulation of co-stimulatory molecules [5], [19], and generation of tolerogenic APCs [20]. In our suppression assays, the CD8^+^CD28^−^ T cells generated by donor APCs plus γc cytokines suppress proliferation of CD4^+^ T cells in a donor-specific and dose-dependent manner. Interestingly, distinct from CD4 Treg cells, the CD8^+^CD28^−^ T cells do not have the ability to suppress proliferation driven by non-specific stimulation by anti-CD3 and anti-CD28 coated Dynabeads. This finding indicates the importance of the interacting APCs in mediating the suppression by the CD8^+^CD28^−^ T cells. Further studies characterizing phenotypic and functional changes of the interacting APCs will provide definitive evidence for this hypothesis. Furthermore, the transwell assay showed that separation of the CD8^+^CD28^−^ T cells alone or CD8^+^CD28^−^ T cells together with stimulator APCs from the responder CD4^+^ T cells abolished the ability of the CD8^+^CD28^−^ T cells to suppress. These data further indicate that suppression of CD8^+^CD28^−^ T cells is mediated through cell-cell contact in a three-cell system, i.e. not only between the CD8^+^CD28^−^ T cells and the APCs, but also between the APCs (or the CD8^+^CD28^−^ T cells) and the responding CD4^+^ T cells.

The finding that suppression by the CD8^+^CD28^−^ T cells is restricted by HLA class I but is promiscuous towards HLA class II specificities with which the responding CD4 TCRs interact suggests a potential mechanism for linked suppression. Linked suppression classically depicts a situation in which co-expression of an indifferent antigen and a tolerant antigen on the same graft allows acceptance of the indifferent antigen in a host previously tolerized to the tolerant antigen [39]. Dendritic cells are thought to play a critical role in the process of linked suppression [39]. In our studies, C-_APC_ cells co-express both matched HLA class I antigens with the priming donor B-_APC_ and mismatched HLA class II antigens with the priming donor B-_APC_. Therefore linked suppression by the CD8^+^CD28^−^ T cells occurs via C-_APC_ cells, and proliferation of CD4^+^ responder to the mismatched class II antigens is suppressed. These findings have significant implications for designing and understanding the therapeutic value of this CD8^+^ suppressor population.

The role of cytotoxicity in mechanisms of regulation by CD8^+^ suppressor cells has been debated. Suppressive CD8^+^CD28^−^ Ts cells generated by repeated stimulations with alloantigens have been shown to have no cytotoxicity towards the stimulating allogeneic APCs [5]. In contrast, Zheng et al [37] reported that human CD8^high^ T cells induced by allogeneic CD40-activated B cells have regulatory property that is at least in part mediated through cytotoxicity towards the allogeneic priming PBMC. In our *in vitro* CFSE-based cytotoxicity assay (Figure 5), it appears that the CD8^+^CD28^−^ T cells generated by our protocol do not possess strong cytotoxicity towards their priming APCs. This is consistent with the down-regulation of perforin in these cells after culturing (Figure 6). It should be noted, however, that cytotoxicity and other possible regulatory mechanisms, such as cytokine production or those mediated through high levels of expression of FoxP3 and CTLA-4, need not be mutually exclusive.

Both cytokine TGF-β [13], [29], [30] and IFN-γ [8], [31], [32] have been implicated in the regulatory mechanisms of CD8^+^ suppressor cells in different studies. In our system, neither anti-IFN-γ nor anti-TGF-β antibody reversed the suppressive effect of the expanded CD8^+^CD28^−^ T cells (Figure 4*C, D*). CD56 and CD57 are two markers of natural killer cells previously reported to over-express on CD8^+^CD28^−^ Ts cells [19]. However, in our culture system the expression of these markers on the CD8^+^CD28^−^ cells decreased after culture, suggesting that they may not play a major role in the functionality of expanded CD8^+^CD28^−^ cells in our system.

Two proteins, FoxP3 and CTLA-4, are discernibly up-regulated in the expanded CD8^+^CD28^−^ T cells. FoxP3 has been described as an important marker for CD8^+^ suppressor cells in humans [18], [20], [40], [41]. However, unlike in mice, in humans non-regulatory T cells including effector cells can also up-regulate FoxP3 expression transiently upon activation [42], [43]. Moreover, there are also CD8^+^ suppressor cells that are not characterized with FoxP3 expression [16], [32], [38]. Whether or not FoxP3 up-regulation in our *in vitro* expanded CD8^+^CD28^−^ T cells contribute to their suppressive function remains to be elucidated. CTLA-4 is another marker described in CD8^+^ suppressor cells [18], [20], [40], [41]. CTLA-4 is a homologue of CD28, and is known as a negative co-stimulatory receptor which upon signaling from CD80 and CD86 delivers inhibitory signals to T cells to down-regulate T cell activation [44], [45]. Recent data also suggest that signaling between CTLA-4 and CD80/CD86 can be bi-directional. CTLA-4 expressed on surface of CD4^+^ Tregs has been shown to interact with CD80/CD86 expressed on APCs and subsequently down-regulate their expression, possibly via a process of trans-endocytosis [46], thereby decreasing the ability of APCs to stimulate T cells through CD28 [47], [48]. CTLA-4 has also been reported to up-regulate indolamine 2,3 dioxygenase (IDO) in some subsets of APCs as yet another mechanism for regulating T cell responses [49]. Given the profound up-regulation of this molecule (from 0.2% to 78.7%, Figure 6) and simultaneous down-regulation of CD28 in our CD8^+^CD28^−^ T cells, CTLA-4 mediated negative signaling to the interacting APCs is a highly attractive potential mechanism of suppression by this cell population.

While robust suppression is observed from our *in vitro* expanded CD8^+^CD28^−^ T cells, an important issue which remains to be resolved is how these cells would behave *in vivo* upon adoptive transfer. Significant plasticity has been described of CD4^+^CD25^+^FoxP3^+^ T cells. Studies of *in vivo* viability, stability and functionality of the expanded human CD8^+^CD28^−^ T cells in our protocol will necessitate the use of functional humanized mouse models of allogeneic immune responses. Such studies are critical before this approach can be safely translated into clinical applications and are currently being actively pursued in our laboratory.

In conclusion, our findings show that donor APCs plus γc cytokines can be harnessed for efficient *in vitro* expansion of human donor-specific CD8^+^CD28^−^ T suppressor cells in a simple and robust culture system. This approach may hold promise for clinical application of using regulatory T cell-based immunotherapy for tolerance induction in transplantation in the future.

## Materials and Methods

### Ethics Statement

This study was approved by the Northwestern Institutional Review Board and written consent was obtained from the participants.

### Human subjects and HLA typing

Peripheral blood mononuclear cells (PBMCs) were obtained from healthy volunteers. HLA typing was performed by the Northwestern Histocompatibility Laboratory using molecular methods. Donors for T cells and APCs were selected for their HLA- A, B and DR compatibility or incompatibility based on the specific requirements of individual experiments.

### Isolation of PBMCs and cell subsets

PBMCs were isolated from fresh whole blood using Lymphocyte Separation Medium (Mediatech Inc., Manassas, VA) by density gradient centrifugation. CD8^+^ and CD4^+^ T cells were purified from PBMCs using CD8 or CD4 isolation kits (Miltenyi Biotec) through positive selection according to the manufacturer's protocols. Purities for both T cells were confirmed by flow cytometry and were routinely >98%. APCs were isolated from PBMCs by depletion of CD3^+^ cells using CD3 microbeads (Miltenyi Biotech). After culturing of the CD8^+^ cells for indicated number of days, CD28^+^ cells were removed by positive selection using human CD28 MicroBead Kit (Miltenyi Biotech, purity>99%), and the flow-through was collected as CD28^−^ cells (purity>95%). Again, purity of all isolated cells was confirmed by flow cytometry.

### *In vitro* generation and expansion of CD8^+^CD28^−^ cells with allogeneic APCs and γc cytokines

2×10^6^ purified CD8^+^ T cells from individual A were cultured with 1×10^6^ HLA-A, -B, -DR mismatched APCs from individual B in 2 ml culture medium (RPMI-1640 supplemented with 15% normal human blood group AB serum, 2 mM L-glutamine, 10 mM HEPES and 1×antibiotic-antimycotic solution; all from Gibco-BRL, Gaithersburg, MD) supplemented with IL-2 (20 U/ml), IL-7 (50 ng/ml) and IL-15 (50 ng/ml) (PeproTech Inc., Rocky Hill, NJ) in 24-well plates at 37°C in 5% CO_2_. Supplemented culture medium was changed on days 4, 7 and 8 (by replacing 1 ml of the culture medium with fresh medium containing cytokines). Cells in each well were split into two wells on day 6 and harvested on day 9, and the CD28^−^ population was isolated as described above.

### Suppression of donor-specific proliferation by *in vitro* generated CD8^+^CD28^−^ cells

5×10^4^ CFSE labeled purified CD4^+^ T cells from individual A (A-T4) were used as “responders (R)” and stimulated with 5×10^4^ APCs from the original priming donor (individual B; B-_APC_). APCs from an HLA-A,-B,-DR complete-mismatched indifferent donor (I-_APC_) or 1×10^4^ anti-CD3/anti-CD28 Dynabeads (Invitrogen, Carlsbad, CA) were used as third party or non-specific stimulation controls respectively. All cultures were prepared in triplicates and incubated in 96-well U-bottom plates in 37°C 5% CO_2_ incubator. The CD8^+^CD28^−^ cells were added as “suppressor (S)” at S∶R ratios of 0.5∶1, 0.1∶1 and 0.02∶1 (the cell number of “R” was kept constant). CFSE dilution was assessed on day 7 to determine extent of proliferation. In selected experiments, anti-human IFN-γ (eBioscience Inc.), anti-human TGF-β antibody (BioXcell, West Lebanon, NH), or respective isotype controls were added to cultures at concentrations of 1 µg/ml, 10 µg/ml or 100 µg/ml. Parallel cultures were analyzed using ^3^H-thymidine incorporation. 1 µCi ^3^H-thymidine (PerkinElmer, San Jose, CA) was added per well for the last 18 hrs of a 7-day culture, and incorporated radioactivity was measured as counts per minute (CPM) with a Perkin-Elmer scintillation counter.

### Transwell experiments

The lower chambers of 24-well transwell plates were plated with either 3×10^5^ CFSE labeled naïve CD4^+^ T cells from individual A (A-T4), or with A-T4 and 3×10^5^ priming APCs from individual B (B-_APC_) in the presence or absence of 3×10^4^ CD8^+^CD28^−^ cells (total volume 600 µl). The upper chambers were plated with medium only, CD8^+^CD28^−^ cells only, or CD8^+^CD28^−^ cells plus priming APCs (B-_APC_). Cells collected from the lower chamber after 7 days of culture were assessed by FACS for CFSE dilution.

### Cytotoxicity by CD8^+^CD28^−^ cells

CFSE-based cytotoxic assay was set up according to published methods [33] as follows. APCs serving as target cells were labeled with two concentrations of CFSE: high concentration (2.0 µM) for APCs from the priming donor (B-_APC_-CFSE^high^) and low concentration (0.2 µM) for APCs from an HLA-A,-B,-DR complete-mismatched indifferent donor (I-_APC_-CFSE^low^). The *in vitro* generated CD8^+^CD28^−^ cells or their CD8^+^CD28^+^ counterpart were used as putative or control effector cells respectively. 5×10^4^ effector cells were cultured with 5×10^4^ each of B-_APC_-CFSE^high^ and I-_APC_-CFSE^low^ together in 96-well U-bottom plates in triplicate wells. Cultured B-_APC_-CFSE^high^ and I-_APC_-CFSE^low^ in the absence of effector cells were used as controls for spontaneous cytolysis. B-_APC_-CFSE^high^ and I-_APC_-CFSE^low^ were enumerated by FACS after 0, 24, 72 and 120 hrs of culture. The ratios of cell numbers of I-_APC_-CFSE^low^ over B-_APC_-CFSE^high^ in the absence or the presence of effector cells were calculated over time to estimate specific killing of B-_APC_-CFSE^high^.

### Multi-color flow cytometric analysis

The following anti-human antibodies and their isotype controls were used: FITC conjugated anti-CD3 (OKT3), CD4(OKT4), CD8(SK1), CD56(MEM188), CD57(TBO1), CD62L(DREG56), CD127(RDR5), Perforin(dG9), all from eBioscience; FITC conjugated anti-human CTLA-4(A3.4H2.H12, Cedarlane USA, Burlington, NC), CD25(BC96), Granzyme B(GB11, Biolegend, San Diego, CA); PE conjugated anti-human Foxp3(eBioscience, San Diego, CA); ECD conjugated anti-human CD8(SFCI21Thy2D3), PE-cyanin 5 (PC5) conjugated anti-human CD28(28.2), PE-cyanin 7 (PC7) conjugated anti-human CD8(SFCI21Thy2D3, Beckman Coulter, Miami, FL). Surface and intra-cellular staining was performed following manufacturers' recommended protocols. Data was acquired on a 5-color FC500 flow cytometer and analyzed using the CXP program (Beckman-Coulter).

### Statistical analysis

Comparisons were made by paired Student's *t* test. Differences were considered significant if the *P* value was <0.05.
